# Comparative Phylogeography of Mississippi Embayment Fishes

**DOI:** 10.1371/journal.pone.0116719

**Published:** 2015-03-31

**Authors:** Jacob J. D. Egge, Taylor J. Hagbo

**Affiliations:** Department of Biology, Pacific Lutheran University, Tacoma, Washington, United States of America; University of Stellenbosch, SOUTH AFRICA

## Abstract

The Mississippi Embayment is a prominent physiographic feature of eastern North America consisting of primarily lowland aquatic habitats and a fish fauna that is largely distinct from nearby highland regions. Numerous studies have demonstrated that both pre-Pleistocene and Pleistocene events have had a strong influence on the distributions and relationships of highland fishes in eastern North America. However, the extent to which these same events affected Embayment distributed taxa remains largely unexplored. The purpose of this study was to investigate the relative roles of pre-Pleistocene and Pleistocene events in shaping phylogeographic relationships of four stream dwelling fishes in the Mississippi Embayment. Molecular genetic analyses of the mitochondrial gene cytochrome *b* were performed for three ictalurid catfish species (*Noturus miurus*, *n* = 67; *Noturus hildebrandi*, *n* = 93, and *Noturus phaeus*, *n* = 44) and one minnow species (*Cyprinella camura*, *n* = 78), all distributed in tributary streams of the Mississippi Embayment. Phylogenetic relationships and divergence times among haplotypes for each species were estimated using maximum likelihood and Bayesian methods. Phylogenetic analyses recovered 6 major haplotype clades within *N*. *miurus*, 5 within *N*. *hildbrandi*, 8 within *N*. *phaeus*, and 8 within *C*. *camura*. All three *Noturus* species show a high degree of isolation by drainage, which is less evident in *C*. *camura*. A clade of haplotypes from tributaries in the southern portion of the Mississippi Embayment was consistently recovered in all four species. Divergence times among clades spanned the Pleistocene, Pliocene, and Miocene. Novel relationships presented here for *C*. *camura* and *N*. *phaeus* suggest the potential for cryptic species. Pre-Pleistocene and Pleistocene era sea level fluctuations coincide with some divergence events, but no single event explains any common divergence across all taxa. Like their highland relatives, a combination of both pre-Pleistocene and Pleistocene era events have driven divergences among Embayment lineages.

## INTRODUCTION

The geographic distribution of stream dwelling organisms is affected by both contemporary and historical ecological conditions (competition, stream size, flow, turbidity, etc.) as well as patterns of stream connectivity—meaning that in addition to temporal or ecological barriers, geographically proximate populations or species may in fact be isolated by distance. Numerous biogeographic studies have addressed these themes in eastern North American aquatic taxa [1,2,3]. The Mississippi River drainage encompasses a significant portion of the aquatic habitats in eastern North America and is the primary center of diversity for stream dwelling organisms including fishes [4,5]. Much of this diversity is found in three highland regions: the Ozarks, Ouachitas, and Appalachians, whose clear high gradient streams harbor high degrees of endemism. Fish diversity in highland regions has been shaped by a combination of pre-Pleistocene and Pleistocene vicariance and dispersal events [1,6,7,8,9,10,11]. The Mississippi River, particularly the lower Mississippi, serves as an important biogeographic barrier to highland taxa in the Appalachians to the east and the Ozarks and Ouachitas to the west [12]. For highland fishes, this barrier consists of predominantly lowland stream habitat, characterized by turbid low gradient streams.

**Fig 1:**
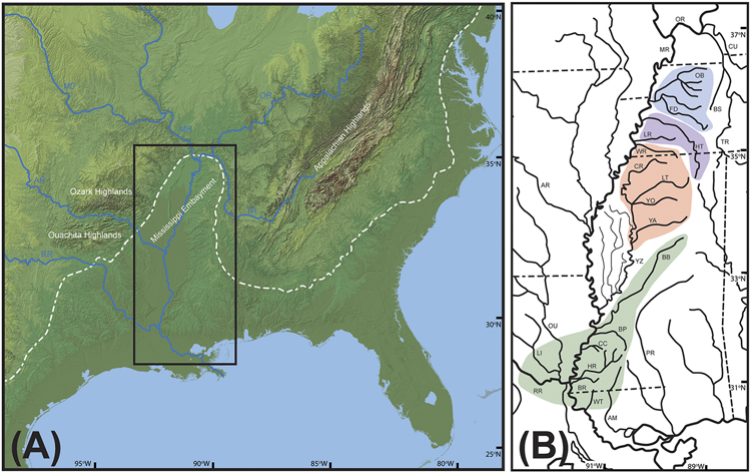
Maps of the Mississippi Embayment region. A. Map of the eastern U.S. illustrating the location of the Mississippi Embayment relative to the Appalachian, Ozark, and Ouachita Highlands. Dashed line represents approximate extent of the Coastal Plain. Black rectangle inset indicates outline of area highlighted in section B. Data available from the U.S. Geological Survey. B. Map of major river drainages in the Mississippi Embayment region. Abbreviations as follows: AM, Amite River; AR, Arkansas River; BB, Big Black River; BP, Bayou Pierre; BR, Buffalo River, BS, Big Sandy River; CC, Coles Creek; CR, Coldwater River; CU, Cumberland River; FD, Forked Deer River; HR, Homochitto River; HT, Hatchie River; LI, Little River; LR, Loosahatchie River; MO, Missouri River; MR, Mississippi River; OB, Obion River; OR, Ohio River; OU, Ouachita River; PR, Pearl River; LT, Little Tallahatchie; RR, Red River; TR, Tennessee River; WR, Wolf River; WT, West Fork Thompson Creek; YA, Yalobusha River; YZ, Yazoo River; YO, Yocona River. Colored regions correspond with color coding used to designate clades in Figs 3–5.

The region encompassing the lower Mississippi River and surrounding lowlands is known as the Mississippi Embayment. The Embayment is an ancient, prominent geomorphological feature of eastern North America. It formed initially as an uplift approximately 95 Ma, but then gradually subsided, separating the once continuous Ouachita-Appalachian mountain range and generating the depressed topography that characterizes the modern day Embayment [13,14,15,16]. The Embayment is effectively an extension of the Coastal Plain, a region characterized largely by lowland rivers that are direct tributaries of the Atlantic Ocean or Gulf of Mexico (Fig 1A). Unlike the rest of the Coastal Plain, most Embayment streams and rivers fall within the Mississippi River drainage. Large rivers with lowland character draining the Ozark and Ouachita highlands dominate the aquatic habitat of the Embayment west of the Mississippi River. In contrast, the eastern side of the Embayment is characterized by a series of small to medium sized rivers and streams that do not drain highland regions, but do contain patches of habitat that have some highland characteristics in their upper reaches (Fig 1B). Some or all of the Embayment was likely influenced by Pleistocene era glacial cycles that correlated with alternating periods of stream aggradation and entrenchment. This process resulted in Embayment streams that were at times more highland in character than they are today [5,17]. Southern tributaries were likely influenced by sea level fluctuations spanning the Miocene, Pliocene, and Pleistocene that periodically resulted in seawater inundation of portions of the Embayment [18].

The ichthyofauna of the Embayment is a mix of lowland species and so-called highland relicts. The lowland fishes generally occupy the large rivers and backwaters in the region including the main channel of the Mississippi River, but some small stream dwelling lowland species are found only in the smaller tributaries. Highland relics comprise a mix of taxa including three minnows (*Cyprinella camura*, *Rhinichthys atratulus* and *Chrosomus erythrogaster*), two darters (*Etheostoma caeruleum* and *Nothonotus rubrus*), one sucker (*Hypentelium nigricans*), and one topminnow (*Fundulus catenatus*). These species are thought to have dispersed into the Embayment during periods of entrenchment in the Pleistocene when some streams were more highland in character [5,17,18,19,20]. Eastern Embayment tributaries contain a number of Embayment endemics including four darters (*Etheostoma raneyi*, *E*. *pyrrhogaster*, *E*. *cervus*, and *Nothonotus rubrus*), two madtom catfishes (*Noturus hildebrandi* and *Noturus phaeus*), and one minnow (*Notropis rafinesquei*).

**Fig 2:**
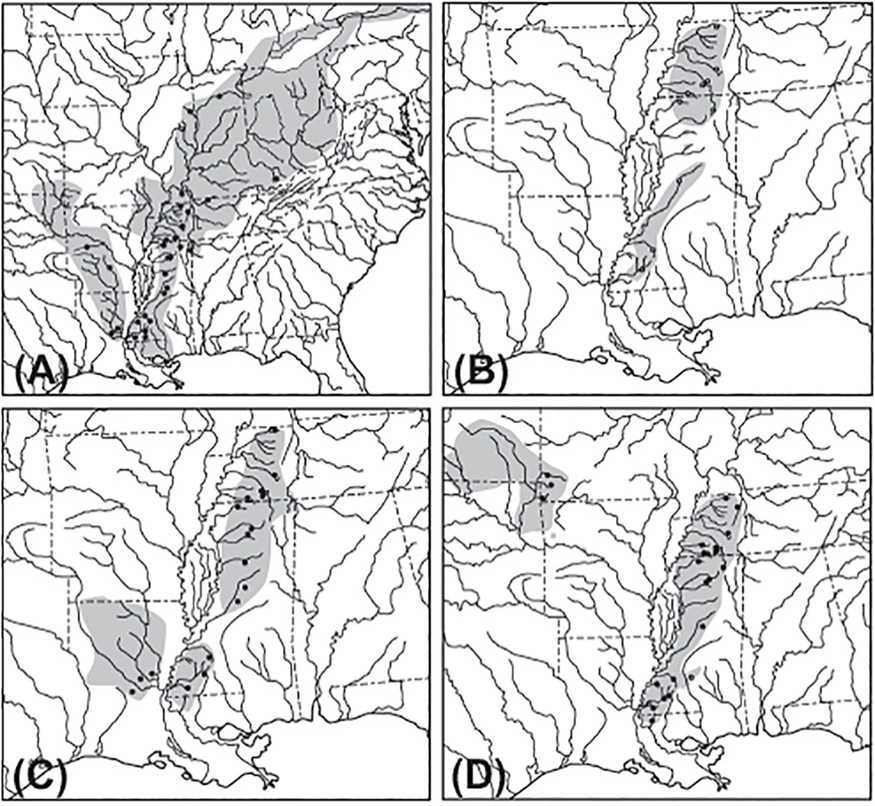
Distribution maps showing sampling localities for each species. A. *Noturus miurus*. B. *Noturus hildebrandi*. C. *Noturus phaeus*. D. *Cyprinella camura*. Gray shading indicates the natural range of each species. Circles indicate approximate sampling localities for specimens acquired for this study (•) and those used in previous studies with sequences acquired from GenBank (◯).

The mix of faunal characteristics, its position as a dispersal barrier between highland regions, and the influence of both glacial cycles and sea level fluctuations make the Embayment a critical region for building a more comprehensive understanding of pattern and process in eastern North American biogeography. The aim of this study was to determine if small stream dwelling fishes widely distributed in the Mississippi Embayment show patterns of Pleistocene dispersal into the Embayment as has been suggested for highland relics. Alternatively, these species may have deeper Embayment origins, consistent with long-term persistence in the region during periods of fluctuating sea levels and drainage rearrangements. In order to determine the extent to which common events shaped the distributions of Embayment distributed species, this study uses phylogenies and divergence time estimates based on mitochondrial DNA sequence data to compare the phylogeographic history of four fish species, three madtom catfishes (Ictaluridae) and one minnow (Cyprinidae), all with broadly sympatric distributions in eastern Embayment streams (Fig 2).

Madtom catfishes (genus *Noturus*) are diminutive members of the family Ictaluridae. Ictalurid species are distributed in both highland and lowland habitats throughout North America east of the Rocky Mountains, however species richness has been shown to be especially high in the eastern Embayment [21]. *Noturus miurus* is the most wide-ranging species examined, extending up the Ohio River drainage into Great Lakes tributaries to the northeast and Arkansas and Ouachita River drainages to the west (Fig 2A). *Noturus hildebrandi* is found only in eastern Embayment tributaries and has the most limited distribution of all four focal species (Fig 2B). *Noturus phaeus* is distributed in small to medium sized streams in both the eastern and parts of the western Embayment (Fig 2C). *Cyprinella camura* a member of the shiner clade [22], a major radiation in the diverse family Cyprinidae. *Cyprinella camura* has a disjunct distribution with western populations found in the upper Arkansas River drainage and eastern populations restricted to eastern tributaries of the Embayment (Fig 2D). Because all of these species are sympatric, their populations have potentially been impacted by similar historic processes operating within the Embayment, making them ideal focal taxa for the goals of this study.

## MATERIALS AND METHODS

### Ethics statement

Field work and collection of specimens was conducted under the following permits: Arkansas Game and Fish Commission Scientific Collecting Permit 040620098, Louisiana Freshwater Scientific Collecting Permit 1306, Mississippi Scientific Collection Permit 0316091, and Tennessee Wildlife Resources Agency Scientific Collection Permit 1501. All procedures with carried out in accordance with a protocol approved by the Pacific Lutheran University Institutional Animal Care and Use Committee (IACUC) approval 09–02.

### Specimen acquisition

Specimens of *N*. *miurus*, *N*. *phaeus*, and *C*. *camura* were collected from all major drainages in the Mississippi Embayment in which they are found (Fig 2). In the case of *N*. *miurus* and *C*. *camura*, additional specimens representing populations found outside of the Embayment were also collected (Fig 2, see S1 Appendix in Supporting Information). Specimens were collected using seines and a backpack electroshocker. Following capture, specimens were euthanized in MS-222 and frozen in liquid nitrogen or preserved directly in 95% ethanol. Voucher specimens for all individuals were deposited in the James Ford Bell Museum Ichthyological Collection (JFBM).

### DNA sequencing and alignment

Muscle tissue or pelvic fin clips were sampled for DNA extraction. DNeasy tissue extraction kits (Qiagen, Valencia, CA, USA) were used to extract genomic DNA following the manufacturer’s protocol. A total of 64 *N*. *miurus*, 43 *N*. *phaeus*, and 76 *C*. *camura* were sequenced for the mitochondrial gene cytochrome *b* (cyt *b* 1138 bp). A comprehensive list of individuals sequenced is provided in S1 Appendix.

The mitochondrial gene cytochrome *b* was amplified using the polymerase chain reaction (PCR) using primers and conditions optimized in previous studies. PCR reactions contained 2.0 μl template DNA, 6.5 μl GoTaq solution (Promega, Madison, WI, USA), 1.0 μl of each primer (10 μM), and 4.5 μl nuclease free H_2_O making a total reaction volume of 15.0 μl. For *Noturus*, the primers GLU-2 and Thr-R1 [23,24] were used for amplification and sequencing. One additional primer, IcCytb-R1, was also used for sequencing [23]. The primers HA(16249) and LA(15058) were used for amplification and sequencing of *Cyprinella* [25]. All reactions were carried out using a Techne Genius thermal cycler (Techne Limited, Oxford, Cambridge, UK) using the following thermal profile: initial denaturation at 94°C (5 min); 35 cycles of 94°C (15 sec); 55°C (15 sec); and 72°C (45 sec); followed by a final extension at 72°C (5 min).

The amplified products were purified using QIAquick PCR purification kits (Qiagen, Valencia, CA, USA) following the manufacturer’s protocols. Sequencing was performed at the University of Washington Department of Biochemistry DNA Sequencing Facility using Big Dye (Perkin Elmer) terminator cycle sequencing on an ABI 3730 XL machine. Sequences were edited, assembled, and aligned by eye using Sequencher 4.0 (Gene Codes) software. All sequences have been deposited in GenBank under Accession numbers KM363003-KM363065 (*N*. *miurus*), KM363066-KM363107 (*N*. *phaeus*), KM363108-KM363183 (*C*. *camura*). Sequences for *N*. *hildebrandi* representing the Obion, Forked Deer, Hatchie, Wolf, Coldwater, Big Black, Bayou Pierre, and Homochitto rivers were acquired as part of a previous study [26] and are deposited in GenBank (S1 Appendix). Additional sequences of *N*. *miurus*, *N*. *phaeus*, and *C*. *camura* as well as outgroup taxa were downloaded from GenBank to supplement the dataset (S1 Appendix).

### Phylogenetic Analyses

Two datasets were created for phylogenetic analyses. One dataset consisted of aligned sequences of all three *Noturus* species. In addition to the focal species, the *Noturus* dataset included outgroup ictalurid taxa representing the genera *Ameiurus*, *Ictalurus*, *Noturus*, *Prietella*, and *Pylodictis* as well as one non-ictalurid, *Cranoglanis bouderius*. A second dataset included all *C*. *camura* sequences in addition to outgroup taxa from the genera *Codoma*, *Cyprinella*, *Dionda* (*Tampichthys*) and *Pimephales* (S1 Appendix).

Best-fit models of sequence evolution and optimal partitioning strategies for each dataset were estimated simultaneously using PartitionFinder [27]. Two separate analyses were performed for each dataset: an unlimited analysis that evaluated all models, and a restricted analysis that considered a limited set of models that could be implemented in MrBayes. The five partitioning strategies evaluated were: 1) three partitions: treat each codon position as a separate partition (partition by codon); 2) two partitions: 1^st^ + 2^nd^ position and 3^rd^ position; 3) two partitions: 2^nd^ + 3^rd^ position and 1^st^ position; 4) two partitions: 1^st^ + 3^rd^ position and 2^nd^ position; and 5) consider the entire gene a single partition. For all searches, the Bayesian information criterion (BIC) was used as the model and partitioning scheme selection criterion.

Gene trees were generated for each dataset using maximum likelihood and Bayesian methods. Partitioned likelihood analyses were implemented in GARLI 2.01 [28] using models and partitioning strategies recovered by the unlimited PartitionFinder analyses. Five independent runs of each dataset with five search replicates each were set to terminate after 10,000 generations of no topology improvement. One hundred bootstrap replicates were generated using identical criteria, except the termination threshold was set to 5,000 generations. Bootstrap values were obtained by compiling the trees and generating a majority-rule consensus topology in PAUP* [29].

Partitioned Bayesian analyses were performed using MrBayes 3.1.2 [30] using models and partitioning strategies recovered by the MrBayes limited PartitionFinder analyses. Two independent Markov chain Monte Carlo (MCMC) simulations were run on each dataset for 5 million generations with four chains (one cold, three heated, *T* = 0.5) sampled every 100 generations using a random starting tree. Convergence was determined graphically and a burnin of 15,000 trees was removed from each analysis. *Cranoglanis bouderius* was used to root the *Noturus* trees and *Pimephales promelas* the *Cyprinella* trees in the Bayesian and likelihood analyses.

### Haplotype network analyses

In order to provide a visualization of haplotype diversity and relationships, statistical parsimony networks of haplotypes were constructed individually for *N*. *miurus*, *N*. *hildebrandi* (inclusive of the *Noturus baileyi* haplotype), *N*. *phaeus*, and *C*. *camura* (inclusive of *Cyprinella galactura* haplotypes) using TCS 1.21 [31].

### Sequence comparisons

Average sequence divergences within and between major clades recovered in the phylogenetic analyses with posterior probabilities >0.95 and bootstrap support >70 were calculated using MEGA 5.2.2 [32]. Both within group mean distances and net between group mean distance options were used to calculate uncorrected pairwise distances. The number of haplotypes and polymorphic sites for major clades were calculated using ARLEQUIN 3.5 [33].

### Divergence time estimates

Tests for constant substitution rates among lineages (molecular clock) were performed using a likelihood ratio test implemented in MEGA 5.2.2 [32]. Molecular clock tests were performed on the same *Noturus* dataset used for Bayesian and likelihood analyses as well as a reduced dataset that included only *Noturus* and *Pylodictis* species. Similarly, a molecular clock test was performed on a reduced *Cyprinella* dataset that included only *C*. *camura*, *C*. *galactura*, and *Cyprinella rutila*. All tests were performed using maximum likelihood topologies generated in MEGA 5.2.2 with models as determined by the PartitionFinder analyses.

Estimates of divergence times among haplotypes were performed using BEAST v1.8.0 [34] implemented on the CIPRES Science Gateway [35]. Previous studies have utilized the relatively rich ictalurid fossil record to establish minimum-age constraints on select nodes to generate divergence time estimates among ictalurid taxa [11,36]. Using fossil references outlined in these studies, the following node age constraints were set using lognormal priors: 1) Ictaluridae, 58 Ma (lognormal mean and standard deviation of 1.1, offset 58) following [36]; 2) *Ictalurus*, 19 Ma (lognormal mean and standard deviation of 1.0, offset 19) following [11] and [36]; 3) *Pylodictis* + *Noturus*, 19 Ma (lognormal mean and standard deviation of 1.0, offset 19) following [36].

Fossil *Noturus* specimens have all been dated to the Pleistocene [37,38,39,40]. Additional specimens designated cf. *Noturus* sp. are known from the Miocene, but are extremely fragmentary and their designation as *Noturus* is tentative [41]. With the exception of the near complete skeleton of *Noturus* cf. *N*. *hildebrandi* from the early Pleistocene [37] and a Weberian apparatus of the same age that might be *Noturus* [38], the specimens consist largely of complete and fragmented pectoral spines. Descriptions of these fossils suggest many of them are likely members of the *rabida* clade (*sensu* [42]) because their pectoral spines are recurved with prominent anterior and posterior serrations. For this reason, a fourth calibration point was set using 2.6 Ma as a minimum age constraint for the *rabida* clade.

The relative paucity and fragmentary nature in the *Noturus* fossil record compared with other ictalurid genera leaves only a single calibration point within the genus. Using fossil calibrations that are much older than the focal taxa, as is the case here, may overestimate node ages [43]. To account for this, we ran separate analyses calibrated using predefined substitution rate estimates. Specific substitution rates for cyt *b* within *Noturus* are unknown. Published fossil calibrated substitution rate estimates for cyt *b* in other teleosts, however, range from 0.76%–2.2% Ma [44,45,46,47,48,49]. Divergence times were estimated under a uniform prior set to encompass the entire range of published cyt *b* substitution rates [9,50]. To minimize the amount of rate heterogeneity in the dataset, these rate calibrated analyses were performed on a reduced dataset consisting only of *Noturus* and *Pylodictis olivaris* sequences. This same procedure was used on a reduced *Cyprinella* dataset that included only *C*. *camura*, *C*. *galactura*, and *C*. *rutila* haplotypes since there are no fossil or biogeographic calibration points to estimate times of divergence in *Cyprinella*.

Fossil and rate calibrated analyses of the *Noturus* dataset and rate calibrated analyses of the *Cyprinella* dataset were performed using the uncorrelated lognormal model (UCLN; [51]) in accordance with the results of the molecular clock tests. Three independent runs were performed for each dataset with MCMC chains run for 50 million generations sampled every 1000 generations. Model parameters for the *Noturus* datasets consisted of a GTR + I + G model partitioned by codon with a lognormal relaxed clock, speciation birth-death process, and random starting tree. In each case, the monophyly of Ictaluridae was enforced. Identical model parameters were set for the *Cyprinella* dataset except a TrN + G model partitioned by codon was set as determined by a PartitionFinder analysis of the reduced dataset.

Results from each run were visualized in Tracer v1.5 [34] to ensure sufficient effective sample sizes, verify convergence of parameter values, and evaluate burn-in for each run prior to stationarity. Samples taken before each run reached stationarity were discarded as burnin. Resulting log and tree files were combined using LogCombiner v1.8.0 [34] with a resampling frequency of 12,000 for the *Noturus* runs. The posterior probability density of the combined tree and log files were summarized with TreeAnnotator v1.8.0 [34].

## RESULTS

### Sequence comparisons

The aligned *Noturus* dataset contained 237 cyt *b* haplotypes (1138 bp) for 67 *N*. *miurus*, 93 *N*. *hildebrandi*, and 44 *N*. *phaeus* and 33 other sequences representing 33 outgroup taxa (S1 Appendix). The aligned *Cyprinella* dataset contained 104 cyt *b* haplotypes (1114 bp) for 78 *C*. *camura*, five *C*. *galactura*, and 21 other sequences representing 15 outgroup taxa (S1 Appendix). The first 15 bp and last 12 bp of the total 1141 bp in the cyt *b* gene were trimmed from the final data matrix because they were missing from most sequences.

The number of unique haplotypes, number of polymorphic sites recovered from each major clade, and average within-clade sequence divergences for each species are presented in Table 1. Pairwise sequence divergences ranged from 0.0000–0.0749 (0.0349 average) in *N*. *miurus*, 0.000–0.0821 (0.0394 average) in *N*. *hildebrandi* (exclusive of *N*. *baileyi*), 0.0000–0.0908 (0.0417 average) in *N*. *phaeus*, and 0.0000–0.0657 (0.0221 average) in *C*. *camura* (exclusive of *C*. *galactura*). Average between-clade sequence divergences for each species are presented in Table 2.

**Table 1:** Sample size (*n*), number of haplotypes, number of polymorphic sites, and average within-clade sequence divergence for all major clades recovered in the phylogenetic analyses.

Species	Clade	*n*	Number of haplotypes	Number of polymorphic sites	Average within-clade sequence divergence
*N*. *miurus*					
	MI	34	22	33	0.0051
	MII	9	5	5	0.0014
	MIII	8	5	3	0.0051
	MIV	6	5	20	0.0069
	MV	6	3	2	0.0016
	MVI	4	1	0	0.0000
*N*. *hildebrandi*					
	HI	21	4	2	0.0015
	HII	21	2	0	0.0012
	HIII	10	2	1	0.0003
	HIV	20	8	8	0.0022
	HV	22	5	15	0.0023
*N*. *phaeus*					
	PI	4	2	18	0.0064
	PII	5	2	2	0.0009
	PIII	10	4	4	0.0035
	PIV	5	2	3	0.0006
	PV	4	1	0	0.0000
	PVI	6	4	5	0.0025
	PVII	7	1	0	0.0000
	PVIII	3	2	1	0.0000
*C*. *camura*					
	CI	5	4	4	0.0020
	CII	14	4	3	0.0005
	CIII	5	2	2	0.0010
	CIV	17	6	6	0.0016
	CV	5	4	5	0.0015
	CVI	4	1	8	0.0000
	CVII	17	8	17	0.0022
	CVIII	11	8	12	0.0035

**Fig 3:**
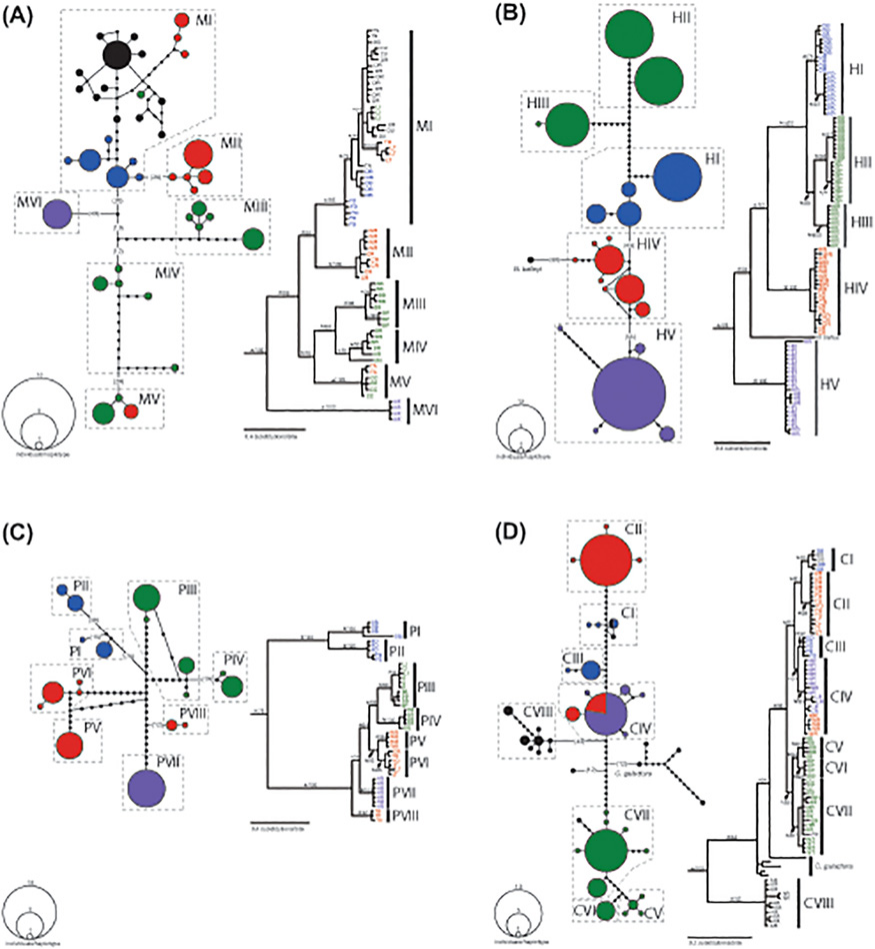
Haplotype networks (at left for each species) and Bayesian consensus topologies (at right for each species) based on cytochrome *b* sequence data. A. *Noturus miurus*. B. *Noturus hildebrandi*. C. *Noturus phaeus*. D. *Cyprinella camura*. Parenthetical numbers indicated haplotypes separated by ≥10 mutational steps. Nodes on trees with posterior probabilities ≥0.95 are indicated with an *. Numbers above nodes indicate likelihood bootstrap support. Locality abbreviations and colors correspond with Fig 1B. Outgroups have been removed from phylogenies for clarity.

**Fig 4:**
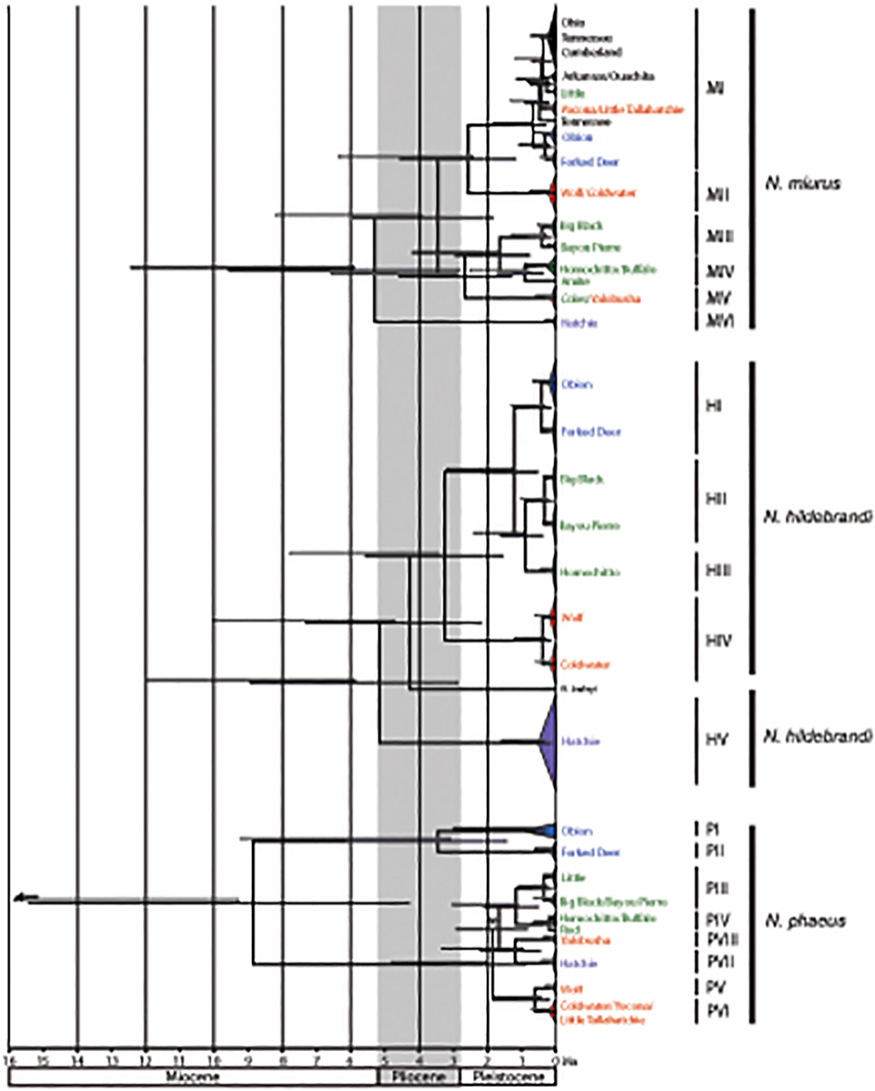
Chronogram for three *Noturus* species estimated from the combined rate-calibrated BEAST analyses based on cytochrome *b* sequence data. Dark bars on nodes represent the 95% highest posterior density of node ages recovered in the rate-calibrated analyses while light bars above nodes represent the same for fossil-calibrated analyses. * indicates the node was not recovered in the fossil-calibrated analyses. Colors correspond with those in Fig 1B. Outgroups removed for clarity.

**Fig 5:**
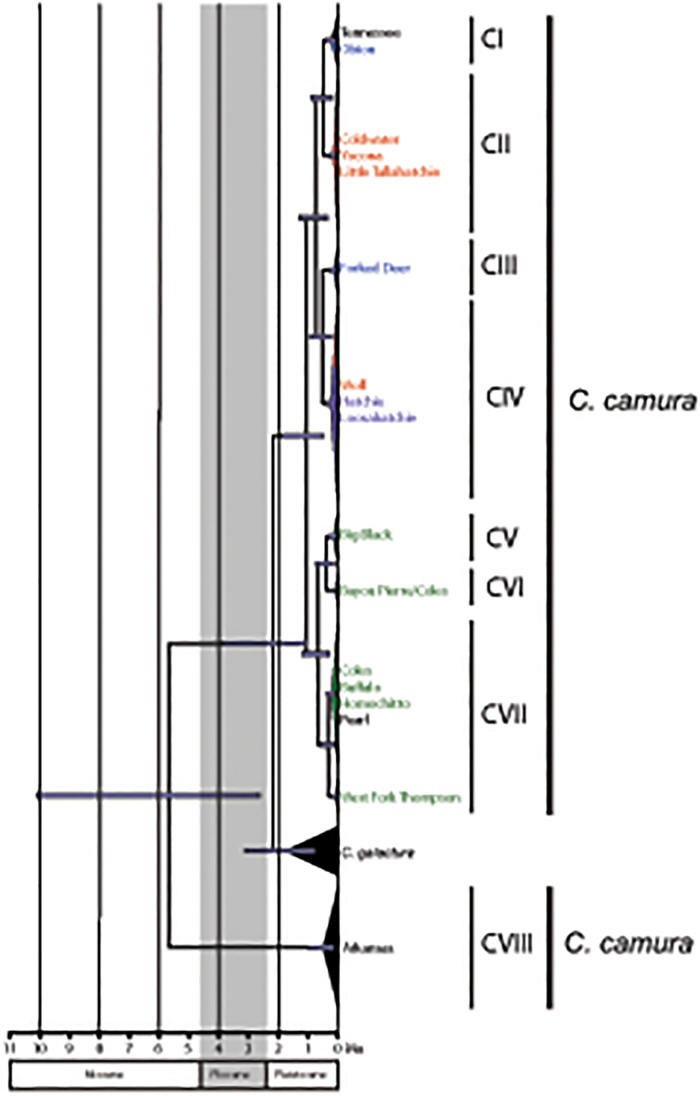
Chronogram for *Cyprinella camura* estimated from the combined BEAST analyses based on cytochrome *b* sequence data. Outgroup (*C*. *rutila*) not shown. Dark bars on nodes represent the 95% highest posterior density of node ages. Colors correspond with those in Fig 1B. Outgroups removed for clarity.

Clade names correspond with those presented in Figs 3–5.

**Table 2:** Average between-clade sequence divergence between major clades recovered in the phylogenetic analyses for each species.

Taxon	Clade/taxon	Average between-clade sequence divergence
*N*. *miurus*		
	MI vs. MII	0.0274
	MIII vs. MIV	0.0182
	MV vs. M(III+IV)	0.0280
	M(I+II) vs. M(III+IV+V)	0.0217
	MVI vs. M(I+II+III+IV+V)	0.0530
*N*. *hildebrandi*		
	HII vs. HIII	0.0139
	HI vs. H(II+III)	0.0120
	HIV vs. H(I+II+III)	0.0429
	*N*. *baileyi* vs. H(I+II+III+IV)	0.0491
	HV vs. *N*. *baileyi*+H(I+II+III+IV)	0.0517
*N*. *phaeus*		
	PI vs. PII	0.0368
	PIII vs. PIV	0.0193
	PV vs. PVI	0.0066
	P(III+IV) vs. P(V+VI)	0.0128
	PVII vs. P(III+IV+V+VI)	0.0133
	PVIII vs. P(III+IV+V+VI+VII)	0.0154
	P(I+II) vs. P(III+IV+V+VI+VII+VIII)	0.0622
*C*. *camura*		
	CI vs. CII	0.0094
	CIII vs. CIV	0.0063
	CV vs. CVI	0.0050
	C(I+II) vs. C(III+IV)	0.0069
	CVII vs. C(V+VI)	0.0056
	C(I+II+III+IV) vs. C(V+VI+VII)	0.0098
	*C*. *galactura* vs. C(I+II+III+IV+VI+VII)	0.0099
	CVIII vs. *C*. *galactura*+C(I+II+III+IV+V+VI+VII)	0.0479

Clade names correspond with those presented in Figs 3–5.

### Phylogenetic Analyses

The best-fit models recovered by PartionFinder are shown for each data partition in Table 3. According to the BIC criterion, the optimal partitioning strategy for both the *Noturus* and *Cyprinella* datasets was to partition by codon in both the unlimited and MrBayes models only analyses. Topologies recovered in the Bayesian and likelihood analyses were largely congruent, although relationships within major clades for each species were generally less well resolved in likelihood analyses. Bayesian consensus topologies are shown for each species individually (excluding outgroups) in Fig 3.

**Table 3:** Best-fit models for cyt *b* data partitions as determined by PartitionFinder analyses using the BIC optimality criterion.

Dataset	Partition	Model	
		Unlimited analysis	MrBayes models only analysis
*Noturus*			
	1^st^ position	TVMef + I + Γ	SYM + I + Γ
	2^nd^ position	TrN + I + Γ	HKY + I + Γ
	3^rd^ position	GTR + I + Γ	GTR + I + Γ
	all positions	GTR + I + Γ	GTR + I + Γ
*Cyprinella*			
	1^st^ position	TrNef + Γ	K80 + I + Γ
	2^nd^ position	TrN + Γ	GTR + Γ
	3^rd^ position	GTR + I + Γ	GTR + Γ
	all positions	GTR + I + Γ	GTR + I + Γ

Results of both an unlimited analysis considering all 56 possible models and a restricted search limited only to models that can be implemented in MrBayes are shown.

Six major clades were recovered within *N*. *miurus* with high posterior probabilities (>0.95) and likelihood bootstrap values (>70) (Fig 3A). Four haplotypes from the Hatchie were recovered as sister to all remaining haplotypes with strong support (posterior probability >0.95, likelihood bootstrap = 100). Well-supported clades largely congruent with individual drainages were recovered for most haplotypes from the Mississippi Embayment. Haplotypes from areas outside the Embayment were all recovered in a single large clade (MI) along with those from the Obion, Forked Deer, Yocona, Little Tallahatchie, and Little River drainages.

Five major clades were recovered within *N*. *hildebrandi* (Fig 3B). *Noturus hildebrandi* cyt *b* haplotypes were found to be non-monphyletic with respect to *N*. *baileyi* as has been reported in previous studies [23,26]. Hatchie haplotypes were recovered as sister to a clade including *N*. *baileyi* and all other *N*. *hildebrandi* haplotypes (Fig 3B).

Eight major clades of *N*. *phaeus* haplotypes were recovered (Fig 3C). The deepest divergence was between the two clades from the Obion and Forked Deer and all other clades. Haplotypes from the Little River were recovered as most closely related to those from the Big Black and Bayou Pierre, a relationship with high posterior (>0.95) and likelihood bootstrap (96) support. Haplotypes from all Yazoo drainages except the Yalobusha were recovered in a pair of well-supported sister clades (PV and PVI; Fig 3C). Nodes supporting the relationships among PIII, PIV, PV, and PVI had likelihood bootstrap values <70, but had posterior probabilities >0.95 (Fig 3C)

Haplotypes from *C*. *camura* were recovered in eight major clades. More broadly, haplotypes from the Mississippi Embayment were recovered in a single well-supported clade (posterior probability >0.95, bootstrap value = 96) while those from the Arkansas drainage were recovered in a separate clade (clade CVIII; Fig 3D). *Cyprinella camura* was not recovered as monophyletic with respect to *C*. *galactura*. Neither likelihood nor Bayeisan analyses recovered *C*. *glactura* haplotypes as monophyletic. However, both analyses recovered *C*. *glactura* haplotypes as more closely related to Embayment *C*. *camura* haplotypes than Arkansas drainage haplotypes (clade CVIII) with strong support (posterior probability >0.95, likelihood bootstrap = 84).

### Haplotype networks

The recovered haplotype networks reflect a general pattern of haplotypes exclusive to specific drainages or regions (Fig 3). Shared haplotypes between drainages were only observed in *N*. *phaeus* and *C*. *camura*. The shared haplotypes among *N*. *phaeus* populations were between the Yocona and Little Tallahatchie and the Buffalo and Homochitto drainages. Shared haplotypes among drainages for *C*. *camura* were found between the Hatchie and Wolf; Yocona, Coldwater, and Little Tallahatchie; Obion and Big Sandy; Bayou Pierre and Coles; and Coles, Buffalo, Homochitto, and Pearl drainages.

### Divergence time estimates

Clock-like substitution rates were rejected for both the full and reduced *Noturus* dataset as well as the full and reduced *Cyprinella* datasets (p<0.05). The estimated 95% highest posterior density (HPD) for the age of clades reconstructed using both fossil-calibrated (FC) and rate-calibrated (RC) methods for the *Noturus* datasets is shown in Fig 4. For complete chronograms including all *Noturus* taxa see S1 and S2 Figs in Supporting Information. Similar 95% HPD estimates for divergence times are shown for the *C*. *camura* dataset in Fig 5. Resulting chronograms were similar in topology to the phylogenies recovered in the likelihood and Bayesian searches (Fig 4). Unlike the phylogenetic analyses, however, Obion and Forked Deer haplotypes of *N*. *miurus* (within clade MI) were recovered as sister clades and the Hatchie and Yalobusha clades (clades PVII and PVIII) of *N*. *phaeus* were recovered in a position sister to clades PIII and PIV. *Cyprinella galactura* haplotypes were also recovered as monophyletic in the chronogram, but not the phylogenetic analyses. In each case, relationships among these clades in likelihood and Bayesian analyses were poorly supported (Fig 3).

Estimated divergence times among *Noturus* clades were consistently older using FC estimates compared with RC estimates. However, the 95% HPD ranges overlap between estimates in all cases (Fig 4; Table 4). The oldest estimated mean divergence time using RC estimates was 8.86 Ma (95% HPD: 4.29–15.42) between *N*. *phaeus* haplotypes from the Obion and Forked Deer (clade PI + PII) and all remaining *N*. *phaeus* haplotypes. FC estimates place this divergence even older with a mean divergence time of 16.29 Ma (95% HPD: 9.29–24.52). Estimated divergence times for Embayment clades of *C*. *camura* were generally younger than for *Noturus* clades (Fig 5), consistent with lower overall sequence divergence between clades (Table 2). The only pre-Pleistocene divergence in the *Cyprinella* dataset was a Miocene age divergence between *C*. *camura* from the Arkansas (clade CVIII) and *C*. *galactura* + *C*. *camura* from the Embayment (mean = 5.67 Ma, 95% HPD: 2.63–10.13).

**Table 4:** Estimated divergence times of common clades recovered in multiple taxa from sister clades in both the rate-calibrated and fossil-calibrated BEAST analyses.

Clade Divergence	Taxon	Mean Age (95% HPD), Ma	
		Rate-calibrated	Fossil-calibrated
Obion + Forked Deer	*N*. *miurus*	0.67 (0.30, 1.20)	1.19 (0.54, 1.83)
	*N*. *hildebrandi*	1.22 (0.55, 2.16)	2.14 (1.25, 3.19)
	*N*. *phaeus*	8.86 (4.29, 15.42)	16.24 (9.29, 24.52)
Wolf + Coldwater	*N*. *miurus*	2.56 (1.18, 4.56)	4.40 (2.41, 6.37)
	*N*. *hildebrandi*	3.26 (1.55, 5.58)	5.51 (3.42, 7.79)
	*N*. *phaeus* ^*1*^	1.85 (0.90, 3.22)	2.61 (1.57, 3.84)
Hatchie	*N*. *miurus*	5.29 (2.80, 9.14)	9.00 (5.91, 12.47)
	*N*. *hildebrandi*	5.16 (2.85, 8.95)	8.89 (5.87, 12.02)
	*N*. *phaeus*	1.19 (0.47, 2.21)	2.10 (0.95, 3.34)
Southern	*N*. *miurus* ^*2*^	3.46 (1.83, 5.92)	4.62 (2.88, 6.58)
	*N*. *hildebrandi*	1.22 (0.55, 2.16)	2.14 (1.25, 3.19)
	*N*. *phaeus*	1.67 (0.83, 2.92)	2.61 (1.57, 3.84)
	*C*. *camura*	0.67 (0.27, 1.22)	—
Big Black + Bayou Pierre	*N*. *miurus*	1.65 (0.77, 2.94)	2.86 (1.62, 4.20)
	*N*. *hildebrandi*	0.90 (0.38, 1.62)	1.56 (0.87, 2.40)
	*N*. *phaeus* ^*3*^	1.17 (0.52, 2.11)	1.99 (1.09, 3.03)
	*C*. *camura*	0.38 (0.13, 0.73)	—

^*1*^ includes Yocona and Little Tallahatchie haplotypes

^*2*^ includes Yalobusha haplotypes

^*3*^ includes Little haplotypes.

## DISCUSSION

### Divergence time estimates

Divergence time estimates for the *Noturus* dataset in this study are similar to those previously reported using both nuclear and mitochondrial loci for ictalurids [11,36] despite the fact that using mtDNA alone has been shown to overestimate divergence times [52,53,54]. While the differences in taxon sampling between studies precludes direct comparison across all nodes, the FC estimated mean origin of *Noturus* in this study was 30.21 Ma (95% HPD: 37.56–59.50), which falls between the 23.9 Ma estimated by [11] and ~37 Ma estimated by [36]. The estimated most recent commons ancestor (MRCA) for *N*. *flavus* and *N*. *gyrinus* was 20.27 Ma (95% HPD: 13.81–26.39) in this study (S1 Fig) which falls between the 16.4 Ma estimated by [11] and ~35 Ma estimated by [36], although these are not sister taxa and topologies differ slightly. RC estimated divergence times at these same nodes were younger than in previous studies, 18.55 Ma (95% HPD: 10.27–31.43) and 12.30 Ma (95% HPD: 6.53–20.84), but had wide 95% HPD estimates (S2 Fig).

Differences between FC and RC divergence estimates could lead to different interpretations regarding the timing and causative explanation of lineage diversification in the *Noturus* dataset. Given the demonstrated tendency for mtDNA and deep fossil calibrations to overestimate node age, the RC estimates, which are consistently younger, are likely the most plausible. For this reason, and the fact that RC estimates were also used to estimate divergence times in the *Cyprinella* dataset, RC estimates will be used in further discussions of *Noturus* divergence times.

### Embayment biogeography—comparative analysis by region

#### Obion and Forked Deer rivers

Haplotypes from the Obion and Forked Deer drainages were consistently recovered in the same clade for all three *Noturus* species. This relationship is consistent with that observed in two darter species endemic to the Obion and Forked Deer drainages: *E*. *pyrrhogaster* from the Obion and its sister species, and *E*. *cervus* from the Forked Deer [55,56]. While the recovery of haplotypes from these drainages in the same clade was consistent, the timing of the divergence of these haplotypes from others differed among species, suggesting that the pattern was not generated by common events (Table 4). The deep divergence between *N*. *phaeus* haplotypes from this region and all others may be indicative of a cryptic species.

In *N*. *miurus* and *C*. *camura* the Obion + Forked Deer clade is not exclusive. In *N*. *miurus* it also includes haplotypes from the Yazoo system, Little River, and localities outside the Embayment including the Tennessee, Cumberland, Ohio, Arkansas, and Ouachita systems. Analyses of lamprey cyt *b* haplotypes for *Lampetra aepyptera* have shown similar close relationship between the Obion and Ohio River drainages [57]. Relationships among other Embayment populations in *N*. *miurus* are characterized by deeper divergences. This pattern is suggestive of *N*. *miurus* originating in the Embayment and greatly expanding its range late in the Pleistocene (Figs 3B and 4), perhaps in a manner similar to highland species dispersing into previously glaciated regions of the Ohio River drainage [8,9]. While other studies have demonstrated or speculated on the movement of highland taxa into the Embayment during the Pleistocene [8,17,20], evidence for Pleistocene dispersal out of the Embayment is novel.

*Cyprinella camura* haplotypes from the Obion and Forked Deer are more broadly included in a clade consisting of haplotypes also from the lower Tennessee, Hatchie, Loosahatchie, Wolf, and Yazoo systems. The presence of a shared haplotype between the Obion and Big Sandy (lower Tennessee system) rivers in *C*. *camura* (Fig 3) suggest recent headwater capture or human mediated introduction may explain the presence of *C*. *camura* in the Big Sandy. Haplotypes from the lower Tennessee system in *N*. *miurus* including Bear Creek and Big Sandy were also recovered as close relatives to those in the Obion, but no shared haplotypes were detected.

#### Hatchie River

For all three *Noturus* species, haplotypes from the Hatchie River form a distinct clade, a result also reported in *L*. *aepyptera* [57]. Perhaps the most striking congruence recovered in this study is the deep near simultaneous divergence between haplotypes from the Hatchie River and all other populations in *N*. *miurus* and *N*. *hildebrandi* (Table 4, Fig 4). The recovery of non-monophyletic *N*. *hildebrandi* cyt *b* hapolotypes with respect to *N*. *baileyi* has been previously reported and is thought to be the result of incomplete lineage sorting or an ancient introgressive event. Multilocus coalescence based species tree analyses of *N*. *hildebrandi* and *N*. *baileyi* suggest that the species are reciprocally monophyletic and that Hatchie River populations are sister to all other *N*. *hildebrandi* populations [26]. Regardless of the species tree relationships, haplotype lineages from the Hatchie in *N*. *miurus* and *N*. *hildebrandi* appear to have undergone near simultaneous divergence in the late Miocene or early Pliocene (Table 4, Fig 4).

Haplotypes of *N*. *phaeus* from the Hatchie River form a distinct clade, but have diverged from other haplotypes more recently in the Pleistocene (Table 4), although their relationship to other clades is poorly supported (Fig 3C). *Cyprinella camura* haplotypes from the Hatchie do not form an exclusive clade and share more recent ancestry with other Embayment haplotypes from the Wolf and Loosahatchie.

#### Wolf and Yazoo rivers

Haplotypes from the Yazoo drainage (Coldwater, Little Tallahatchie, Yocona, and Yalobusha rivers) show varying patterns of relationship to surrounding drainages. This pattern is consistent with the hypothesis that the modern Yazoo is a composite of drainages that were once independent tributaries to an ancient Ohio or Mississippi River, but were also at various times during the Pleistocene connected with the Wolf and Hatchie to the north and possibly other drainages to the south [58].

Haplotypes from the Wolf River were found to be closely related to those from the Coldwater in all three *Noturus* species (Fig 3A–3C). The close relationship between the Wolf and Coldwater haplotypes is consistent with the existence of an ancient Wolf-Coldwater drainage proposed by [58]. While the divergence times between the Wolf and Coldwater clades appear to be similar across *Noturus* species (Fig 4), the timing of divergences for the Wolf + Coldwater clade varies from Pliocene to Pleistocene in age (Table 4, Fig 4). The recovery of haplotypes from the Wolf in a clade with those from the Hatchie and Loosahatchie in *C*. *camura* supports the existence of a proposed Wolf-Hatchie River that drained through the Yazoo basin in the Pleistocene [58].

#### Southern drainages

The recovery of a clade of haplotypes from drainages ranging from the Big Black River south to the West Fork of Thompson Creek was the most consistently recovered clade common to all four species (Fig 3). This pattern suggests a biogeographic divide between the Yazoo and Big Black rivers. As noted by [59], the Yazoo forms the southern range limit of snubnose darters (*Etheostoma*, subgenus Ulocentra), while other species including *Cyprinella whipplei*, *Lythrurus rosepinnis*, and *Notropis texanus* are common on one side of the divide, but not the other [60]. A sister relationship between haplotype clades from the Big Black and Bayou Pierre rivers was consistently recovered in all four species (Fig 3). Divergence time estimates for this clade suggest a degree of pseudocongruence as divergence times vary from mid Pliocene in *N*. *miurus* to late Pleistocene for *C*. *camura* (Table 4; Figs 4 and 5).

Relationships of haplotypes of *N*. *miurus* and *N*. *phaeus* from the west side of the Embayment in the Little and Red drainages suggest that these species dispersed into these drainages in different ways. *Noturus phaeus* haplotypes from the Little and Red drainages are related to those from the Southern clade. Haplotypes from the Little are related to Big Black and Bayou Pierre haplotypes and those from the Red are related to haplotypes from the Homochitto and Buffalo drainages (Fig 3C). This suggests that *N*. *phaeus* individuals from the Southern clade have dispersed across the Mississippi River multiple times. These events may in part have been facilitated by a hypothesized westward shift in the lower Mississippi River from approximately the mouth of the Big Black River south followed by an eastward movement into its current position during the Pleistocene [58], a timeframe consistent with the estimated divergence times of these haplotypes (Fig 4). The same exchange across the Mississippi River did not appear to happen in *N*. *miurus*. Haplotypes of *N*. *miurus* from the Little drainage are most closely related to those from the Arkansas and Ouachita drainages (Fig 3A), suggesting *N*. *miurus* dispersed from elsewhere, perhaps from the north.

The Amite and Pearl rivers are not direct tributaries of the Mississippi, but are geographically proximate to the headwaters of eastern Embayment streams included in the Southern clade. Haplotypes from both *N*. *miurus* (Amite; Fig 3A) and *C*. *camura* (Pearl; Fig 3D) from these drainages suggest historic faunal exchange. This pattern is consistent with other studies that recover close relationships between fish populations in the southern Embayment and coastal drainages [8,59,61].

### Historic climatic factors and Embayment biogeography

Results suggest deep structure among *Noturus* populations within the Embayment and demonstrate that both pre-Pleistocene and Pleistocene era processes are responsible for diversification among populations (Table 2; Table 4; Fig 4). The Mississippi River acts as a contemporary barrier to dispersal for *Noturus* species as demonstrated by a high degree of isolation by drainage. With the exception of *Noturus* haplotypes from the Hatchie, divergence times for most clades defined by individual streams are late Pleistocene in age (<1 Ma) in all four species (Figs 4 and 5), suggesting isolation of populations in individual drainages occurred recently. This pattern is consistent with hypotheses proposed by [58] that suggests eastern tributary streams of the Embayment underwent significant changes in their downstream connectedness as the Mississippi and Ohio rivers migrated to their current course and the modern arrangement of tributaries was set in the late Pleistocene.

Divergence time estimates suggest that *C*. *camura* is a more recent arrival to the Embayment with all divergences occurring in the Pleistocene. A mean divergence time of 5.67 Ma suggests a Miocene age split from other *C*. *camura* populations in the upper Arkansas River followed by a more recent divergence occurring near the end of the Pliocene or early Pleistocene from *C*. *galactura*, a highland distributed relative. Although we cannot rule out the possibility of mitochondrial introgression between *C*. *camura* and *C*. *galactura*, the timing of these divergences would be consistent with *C*. *camura* dispersing into the Embayment during periods in the Pleistocene when streams were more highland in character [5,17,18]. This is consistent with the hypothesized Pleistocene dispersal of other highland relicts into the Embayment including *E*. *caeruleum* [20] and *H*. *nigricans* [8].

Sea level fluctuations have been shown to strongly influence population structure and facilitate speciation in coastal freshwater fishes [62,63,64,65]. Periods of low sea level promote connectedness of river systems as they extend further to reach the coast. This connectedness promotes dispersal among drainages. Subsequent periods of high sea level, conversely, isolate populations and lead to divergence. Previous studies have implicated fluctuating sea levels in the Miocene, Pliocene, and Pleistocene as factors influencing diversification of North American ichthyofauna. While no single fluctuation can explain common divergences across all species in this study, there are several divergences that correspond with fluctuations hypothesized to have influenced diversification in other North American taxa. A dramatic drop in sea level at the Miocene-Pliocene boundary followed by a subsequent dramatic rise in sea level in the Pliocene corresponds with the deep Hatchie clade divergence observed in *N*. *miurus* (5.23 Ma [95% HPD: 2.80–9.14]) and *N*. *hildebrandi*, (5.16 Ma [95% HPD: 2.85–8.95]) (Table 4, Fig 4). This event also corresponds with speciation in black basses, genus *Micropterus* [66], a deep split between eastern and western clades of *Nocomis* species [50], and diversification of a clade-rich and widespread clade of *Noturus exilis* [11].

The dramatic rise in sea level (50–80 m above current levels) lasting for approximately one million years in the Pliocene [67,68,69] has been implicated in the diversification of *L*. *aepyptera* populations in the Embayment [57]. The extent to which seawater inundated the Embayment is unclear, but it likely submerged most of the drainages in the southern Embayment [18]. Southern clade haplotypes of all four species diverge after this event, although not simultaneously, but suggesting that if these species were in this region prior to the Pliocene, their populations would have been extirpated due to seawater inundation (Table 4, Figs 4 and 5). Recolonization of the region would have followed sea level retreat and establishment of freshwater habitats. Divergences between the Wolf + Coldwater clade (HIII) from its sister clade in *N*. *hildebrandi*, the Obion and Forked Deer clades (PI and PII) in *N*. *phaeus*, and the Southern clade from its sister clade in *N*. *miurus* occur later in the Pliocene coincident with another sea level fluctuation. Subsequent fluctuations in the Pleistocene may have also played a role in lineage diversification among the four focal taxa, but no common impacts are apparent.

## Supporting Information

S1 Appendix Material examined. List of voucher specimens used to generate sequences for this study along with corresponding GenBank accession numbers. Additional sequences generated in previous studies acquired from GenBank are listed below. JFBM = James Ford Bell Museum Ichthyological Collection.
(DOCX)

S1 Fig Fossil-calibrated *Noturus* chronogram. Fossil-calibrated chronogram inclusive of all ictalurid taxa estimated from the combined BEAST analyses based on cytochrome *b* sequence data. Bars on nodes represent 95% highest posterior density of node ages. Clade names correspond with those in Figs 3 and 4.
(PDF)

S2 Fig Rate-calibrated *Noturus* chronogram. Rate-calibrated chronogram inclusive of all ictalurid taxa estimated from the combined BEAST analyses based on cytochrome *b* sequence data. Bars on nodes represent 95% highest posterior density of node ages. Clade names correspond with those in Figs 3 and 4.
(PDF)
