# Correction: Comparative Phylogeography of Mississippi Embayment Fishes

**DOI:** 10.1371/journal.pone.0128725

**Published:** 2015-05-26

**Authors:** Jacob J. D. Egge, Taylor J. Hagbo

Figs 2–5 have been corrected for improved readability. Please see the corrected Fig 2 here.

**Fig 2:**
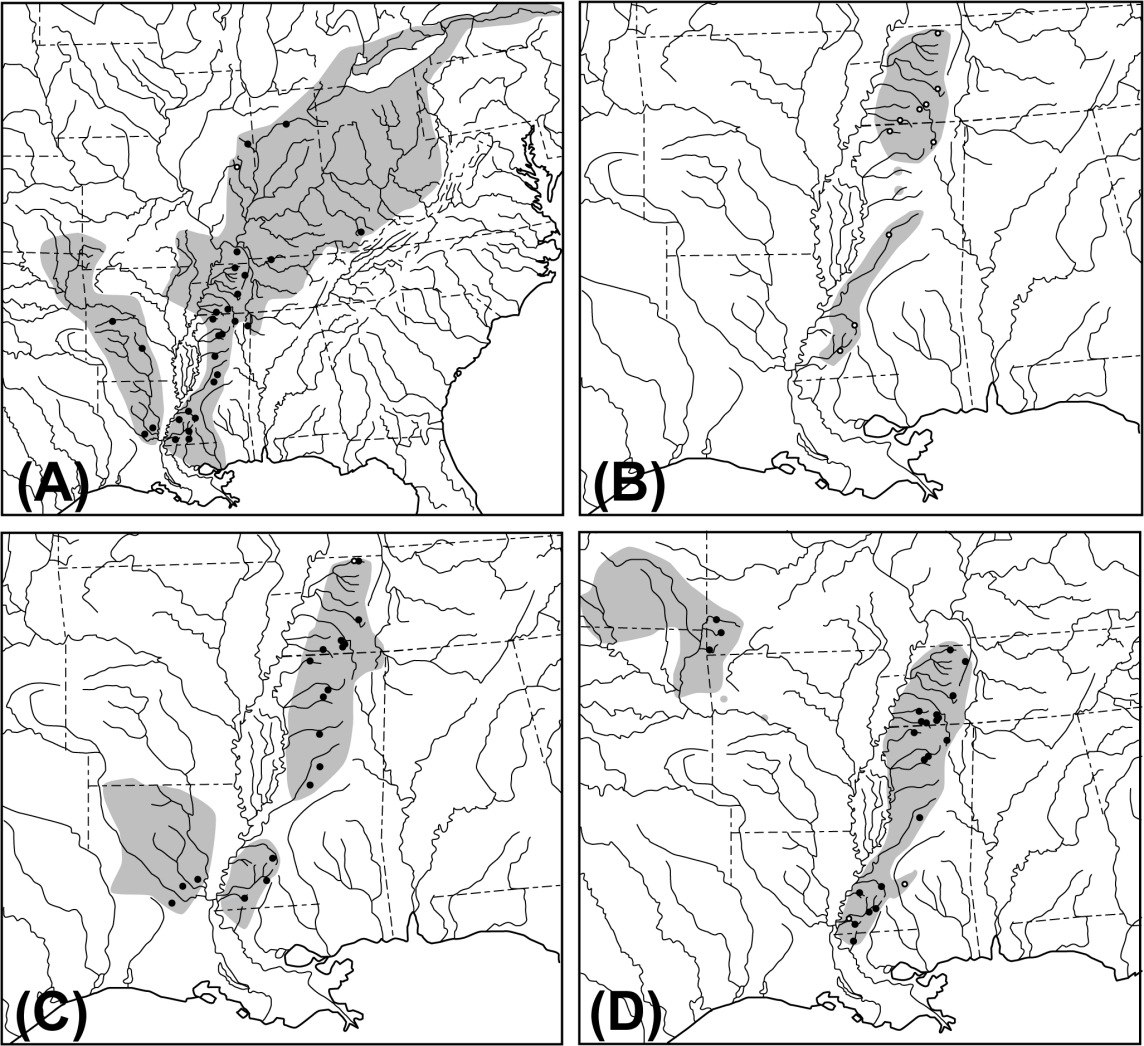
Distribution maps showing sampling localities for each species. A. *Noturus miurus*. B. *Noturus hildebrandi*. C. *Noturus phaeus*. D. *Cyprinella camura*. Gray shading indicates the natural range of each species. Circles indicate approximate sampling localities for specimens acquired for this study (•) and those used in previous studies with sequences acquired from GenBank (◯).

Please see the corrected Fig 3 here.

**Fig 3:**
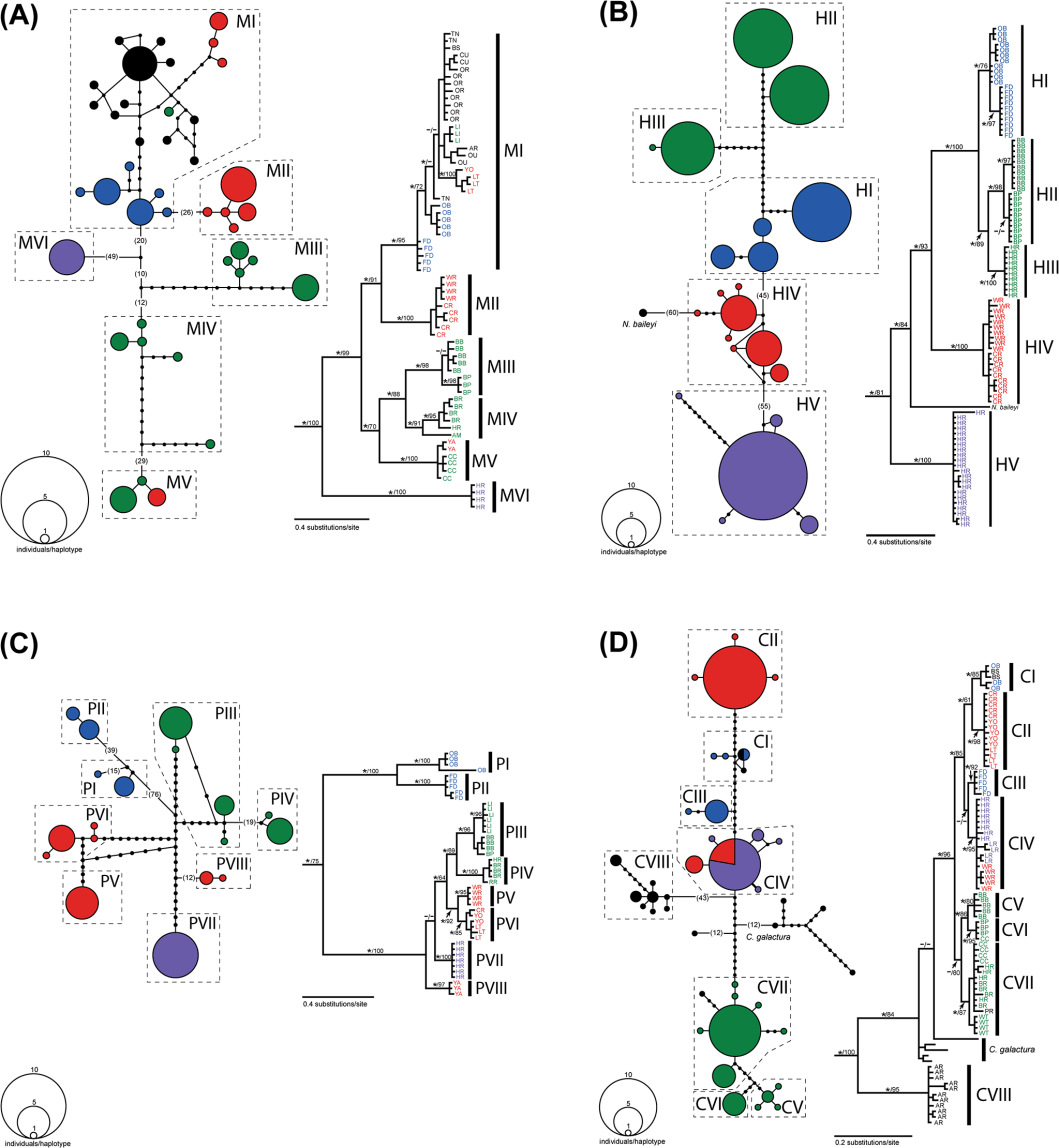
Haplotype networks (at left for each species) and Bayesian consensus topologies (at right for each species) based on cytochrome *b* sequence data. A. *Noturus miurus*. B. *Noturus hildebrandi*. C. *Noturus phaeus*. D. *Cyprinella camura*. Parenthetical numbers indicated haplotypes separated by ≥10 mutational steps. Nodes on trees with posterior probabilities ≥0.95 are indicated with an *. Numbers above nodes indicate likelihood bootstrap support. Locality abbreviations and colors correspond with Fig 1B. Outgroups have been removed from phylogenies for clarity.

Please see the corrected Fig 4 here.

**Fig 4:**
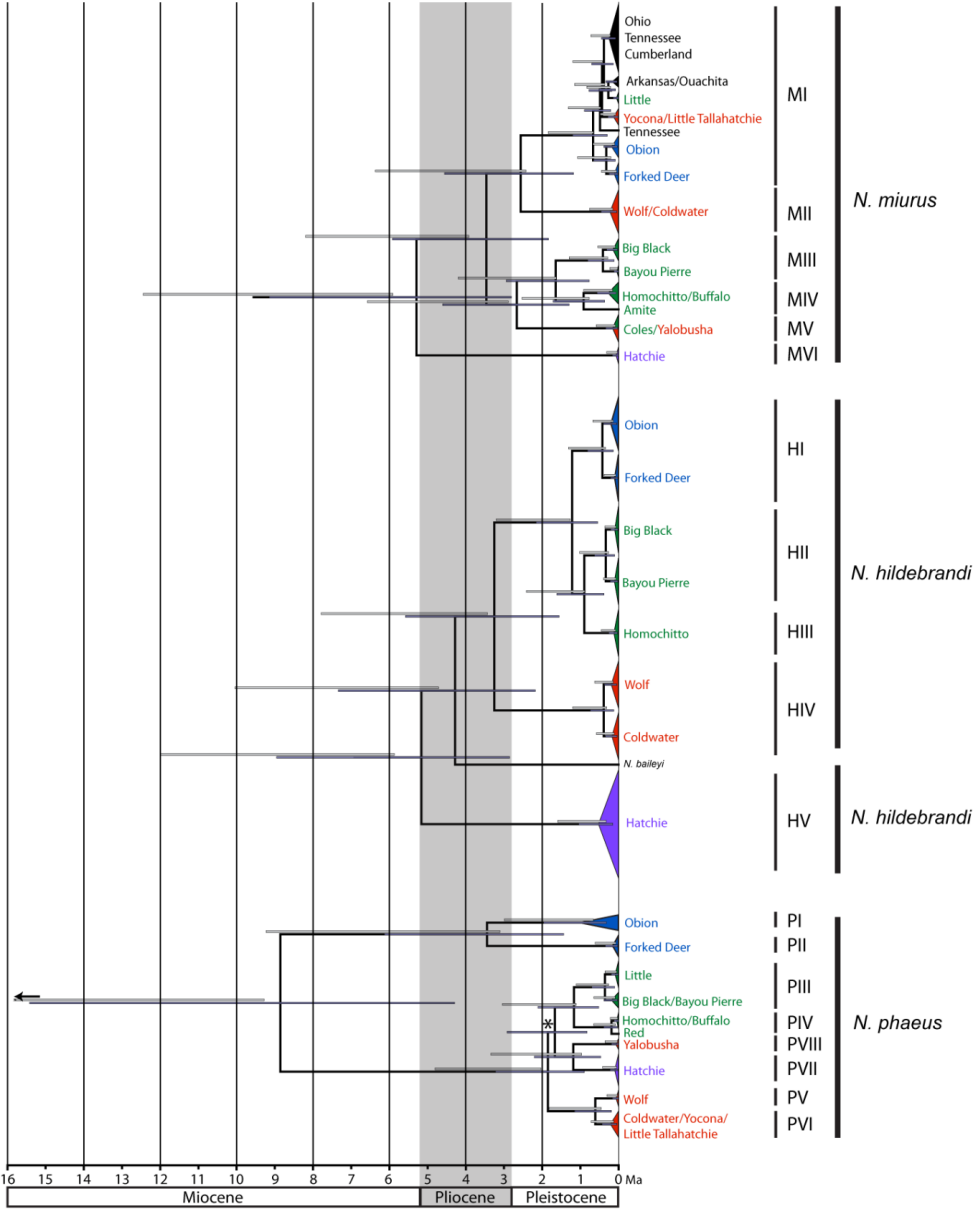
Chronogram for three *Noturus* species estimated from the combined rate-calibrated BEAST analyses based on cytochrome *b* sequence data. Dark bars on nodes represent the 95% highest posterior density of node ages recovered in the rate-calibrated analyses while light bars above nodes represent the same for fossil-calibrated analyses. * indicates the node was not recovered in the fossil-calibrated analyses. Colors correspond with those in Fig 1B. Outgroups removed for clarity.

Please see the corrected Fig 5 here.

**Fig 5:**
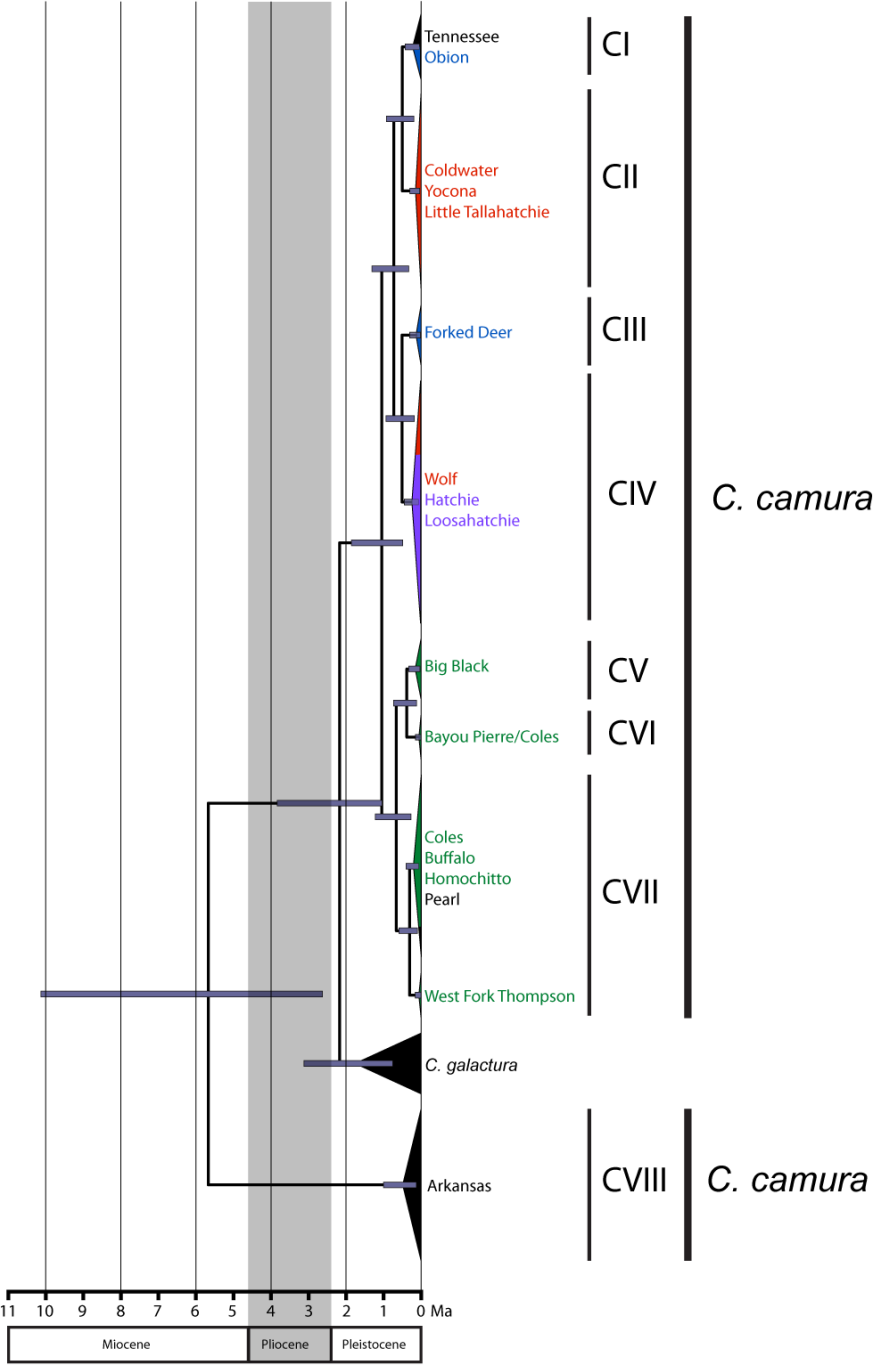
Chronogram for *Cyprinella camura* estimated from the combined BEAST analyses based on cytochrome *b* sequence data. Outgroup (*C*. *rutila*) not shown. Dark bars on nodes represent the 95% highest posterior density of node ages. Colors correspond with those in Fig 1B. Outgroups removed for clarity.
