# Supplementary material for: Comparative Phylogeography of Mississippi Embayment Fishes
Source: PLoS One. 2015 Mar 31;10(3):e0116719. doi: 10.1371/journal.pone.0116719 (PMC4380359; doi:10.1371/journal.pone.0116719)
Supplement: S1 Appendix — List of voucher specimens used to generate sequences for this study along with corresponding GenBank accession numbers. Additional sequences generated in previous studies acquired from GenBank are listed below. JFBM = James Ford Bell Museum Ichthyological Collection. (DOCX) [file pone.0116719.s001.docx]

Appendix S1. List of voucher specimens used to generate sequences for this study along with corresponding GenBank accession numbers. Additional sequences generated in previous studies acquired from GenBank are listed below. JFBM = James Ford Bell Museum Ichthyological Collection. State abbreviations as follows: AR = Arkansas, IL = Illinois, IN = Indiana, KY = Kentucky, LA = Louisiana, TN = Tennessee, MO = Missouri, MS = Mississippi.

| Species | Drainage | Locality | *n* | Catalog # | GenBank  Accession # |
| --- | --- | --- | --- | --- | --- |
| *Cyprinella camura* | Obion | Clear Creek; Henry Co., TN | 3 | JFBM 47746 | KM363108-KM363110 |
|  | Forked Deer | Clark’s Creek; Chester Co., TN | 5 | JFBM 47747 | KM363113-KM363117 |
|  | Hatchie | Pleasant Run; Hardeman Co., TN | 2 | JFBM 47748 | KM363118-KM363119 |
|  |  | Spring Creek; Hardeman Co., TN | 2 | JFBM 47749 | KM363122-KM363123 |
|  |  | East Fork Spring Creek; Hardeman Co., TN | 2 | JFBM 47750 | KM363124-KM363125 |
|  |  | Hatchie River; Tippah Co., MS | 2 | JFBM 47751 | KM363120-KM363121 |
|  | Loosahatchie | Loosahatchie River; Fayette Co., TN | 4 | JFBM 47752 | KM363126-KM363129 |
|  | Wolf | Wolf River; Shelby Co., TN | 4 | JFBM 47753 | KM363130-KM363133 |
|  |  | Wolf River; Shelby Co., TN | 1 | JFBM 47754 | KM363134 |
|  | Coldwater (Yazoo) | Coldwater River; Marshall Co., MS | 5 | JFBM 47755 | KM363135-KM363139 |
|  | Little Tallahatchie (Yazoo) | Puskus Creek; LaFayette Co., MS | 5 | JFBM 47756 | KM363140-KM363144 |
|  | Yocona (Yazoo) | Pumpkin Creek; LaFayette Co., MS | 4 | JFBM 47757 | KM363145-KM363148 |
|  | Big Black | Little Zilpha Creek; Attala Co., MS | 5 | JFBM 47758 | KM363149-KM363153 |
|  | Bayou Pierre | Bayou Pierre; Copiah Co., MS | 3 | JFBM 47759 | KM363154-KM363156 |
|  | Coles Creek | North Fork Coles Creek; Jefferson Co., MS | 5 | JFBM 47760 | KM363157-KM363161 |
|  | Homochitto | Brushy Creek; Amite Co., MS | 3 | JFBM 47761 | KM363162-KM363164 |
|  |  | Horse Creek; Franklin Co., MS | 1 | JFBM 47762 | KM363168 |
|  | Buffalo | Buffalo River; Wilkinson Co., MS | 3 | JFBM 47763 | KM363165-KM363167 |
|  | West Fork Thompson | West Fork Thompson Creek; East Feliciana/West Feliciana Parish, LA | 4 | JFBM 47764 | KM363169-KM363172 |
|  | Big Sandy (Tennessee) | Martin Creek; Carroll Co., TN | 2 | JFBM 47765 | KM363111-KM363112 |
|  | Arkansas | Elk River; McDonald Co., MO | 9 | JFBM 46429 | KM363175-KM363183 |
|  |  | Spring River; Jasper Co., MO | 1 | JFBM 42091 | KM363173 |
|  |  | Shoal Creek; Newton Co., MO | 1 | JFBM 46880 | KM363174 |
|  |  |  |  |  |  |
|  |  |  |  |  |  |
| Species | Drainage | Locality | *n* | Catalog # | GenBank  Accession # |
| *Noturus miurus* | West Fork Clark’s | West Fork Clark’s River; Graves Co., KY | 1 | uncataloged | KM363052 |
|  | Obion | Clear Creek; Henry Co., TN | 4 | JFBM 40910 | KM363003-KM363006 |
|  | Forked Deer | Clark’s Creek; Chester Co., TN | 2 | JFBM 42701 | KM363010-KM363011 |
|  |  | Clark’s Creek; Chester Co., TN | 4 | JFBM 47728 | KM363007-KM363009 |
|  | Hatchie | East Fork Spring Creek; Hardeman Co., TN | 1 | JFBM 47729 | KM363012 |
|  |  | Spring Creek; Hardeman Co., TN | 2 | JFBM 47776 | KM363014-KM363015 |
|  |  | Hatchie River; Tippah Co., MS | 1 | JFBM 47730 | KM363013 |
|  | Wolf | Wolf River; Fayette Co., TN | 3 | JFBM 37539 | KM363016-KM363018 |
|  | Coldwater (Yazoo) | Coldwater River; DeSoto Co., MS | 2 | JFBM 47777 | KM363019-KM363020 |
|  |  | Coldwater River; DeSoto Co., MS | 3 | JFBM 47331 | KM363021-KM363023 |
|  | Little Tallahatchie (Yazoo) | Puskus Creek; LaFayette Co., MS | 3 | JFBM 47732 | KM363025-KM363027 |
|  | Yocona (Yazoo) | Pumpkin Creek; LaFayette Co., MS | 1 | JFBM 47733 | KM363024 |
|  | Yalobusha (Yazoo) | Horse Pen Creek; Calhoun Co., MS | 2 | JFBM 47734 | KM363028-KM363029 |
|  | Big Black | Little Zilpha Creek; Attala Co., MS | 3 | JFBM 47735 | KM363032-KM363034 |
|  |  | Long Creek; Attala Co., MS | 2 | JFBM 47736 | KM363030-KM363031 |
|  | Bayou Pierre | Bayou Pierre; Copiah Co., MS | 2 | JFBM 47737 | KM363035-KM363036 |
|  |  | Bayou Pierre; Copiah Co., MS | 1 | JFBM 47738 | KM363037 |
|  | Coles Creek | North Fork Coles Creek; Jefferson Co., MS | 4 | JFBM 37463 | KM363038-KM363041 |
|  | Homochitto | Horse Creek; Franklin Co., MS | 1 | JFBM 47739 | KM363046 |
|  | Buffalo | Buffalo River; Wilkinson Co., MS | 4 | JFBM 47740 | KM363042-KM363045 |
|  | Amite | East Fork Amite River; Amite Co., MS | 1 | JFBM 47741 | KM363047 |
|  | Tennessee | Bear Creek; Tishomingo Co., MS | 2 | JFBM 47778 | KM363053-KM363054 |
|  | Big Sandy (Tennessee) | Martin Creek; Carroll Co., TN | 1 | JFBM 47742 | KM363055 |
|  | Cumberland | Red River; Montgomery/Robertson Co., TN | 2 | JFBM 47779 | KM363056-KM363057 |
|  | Wabash (Ohio) | Tippecanoe River; Fulton Co., IN | 4 | JFBM 43434 | KM363059-KM363062 |
|  |  | Middle Fork Vermillion River; Vermillion Co., IL | 1 | JFBM 47780 | KM363063 |
|  | Ohio | Red Bird River; Clay Co., KY | 2 | JFBM 45582 | KM363064-KM363065 |
|  | Arkansas | Fourche LaFave River; Yell Co., AR | 1 | JFBM 47743 | KM363049 |
|  | Little | Sandy Creek; Grant Parish, LA | 1 | JFBM 47744 | KM363048 |
|  |  | Greens Creek; Catahoula Parish, LA | 2 | JFBM 47745 | KM363050-KM363051 |
|  | Ouachita | Saline River; Grant Co., AR | 1 | JFBM 42655 | KM363058 |
|  |  |  |  |  |  |
| *Noturus phaeus* | Obion | Clear Creek; Henry Co., TN | 3 | JFBM 40911 | KM363066-KM363068 |
|  | Forked Deer | Clark’s Creek; Chester Co., TN | 5 | JFBM 42702 | KM363069-KM363073 |
|  | Hatchie | East Fork Spring Creek; Hardeman Co., TN | 2 | JFBM 44235 | KM363076-KM363077 |
|  |  | Spring Creek; Hardeman Co., TN | 3 | JFBM 47782 | KM363078-KM363080 |
|  |  | Pleasant Run; Hardeman Co., TN | 2 | JFBM 42728 | KM363074-KM363075 |
| Species | Drainage | Locality | *n* | Catalog # | GenBank  Accession # |
| *Noturus phaeus* (cont.) | Wolf | Wolf River; Fayette Co., TN | 4 | JFBM 37541 | KM363081-KM363084 |
|  | Coldwater (Yazoo) | Coldwater River; Marshall Co., MS | 1 | JFBM 44109 | KM363085 |
|  | Little Tallahatchie (Yazoo) | Puskus Creek; LaFayette Co., MS | 3 | JFBM 47766 | KM363087-KM363089 |
|  | Yocona (Yazoo) | Pumpkin Creek; LaFayette Co., MS | 1 | JFBM 47435 | KM363086 |
|  | Yalobusha (Yazoo) | Horse Pen Creek; Calhoun Co., MS | 3 | JFBM 47768 | KM363090-KM363092 |
|  | Big Black | Little Zilpha Creek; Attala Co., MS | 1 | JFBM 47769 | KM363093 |
|  |  | Long Creek; Attala Co., MS | 2 | JFBM 47770 | KM363094-KM363095 |
|  | Bayou Pierre | Brushy Creek; Copiah Co., MS | 1 | JFBM 47771 | KM363096 |
|  | Homochitto | Horse Creek; Franklin Co., MS | 1 | JFBM 47772 | KM363097 |
|  | Buffalo | Buffalo River; Wilkinson Co., MS | 3 | JFBM 47773 | KM363098-KM363100 |
|  | Little | Sandy Creek; Grant Parish, LA | 2 | JFBM 47774 | KM363101-KM363102 |
|  |  | Greens Creek; Catahoula Parish, LA | 4 | JFBM 47775 | KM363103-KM363106 |
|  | Red | Spring Creek; Rapides Parish, LA | 1 | JFBM 47767 | KM363107 |

Sequences of focal taxa downloaded from GenBank are as follows: *C. camura* (GQ275234, GQ275235, AY327315); *N. hildebrandi* (DQ790746, AY327299, AY327300, AY327298, KM264017-KM264126); *N. miurus* (DQ790738, AY327306, DQ790739, KM264123); and *N. phaeus* (AY327315, KM264124).

GenBank accession numbers for outgroup taxa used in the *Noturus* dataset are as follows: *Ameiurus brunneus* (AY184260); *A. catus* (AF484163); *A. natalis* (AY184265); *Ictalurus furcatus* (AF484159); *I. punctatus* (AY184253); *Noturus albater* (AY327268); *N. crypticus* (DQ383659); *N. elegans* (AY327274); *N. eleutherus* (AY327278); *N. exilis* (AY327325); *N. fasciatus* (AY327276); *N. funebris* (AY327291); *N. gilbert* (AY327294); *N. gladiator* (AY327319); *N. gyrinus* (AY327295); *N. flavater* (AY327283); *N. flavipinnis* (AY327284); *N. flavus* (AY327287); *N. furiosus* (AY327292); *N. insignis* (AY327301); *N. lachneri* (AY327304); *N. leptacanthus* (AY327305); *N. maydeni* (AY327271); *N. munitus* (AY327309); *N. nocturnus* (AY327311); *N. placidus* (AY327318); *N. stanauli* (DQ383661); *N. stigmosus* (AY327320); *N. taylori* (AY327322); *Prietella phreatophila* (AY458884); *Pylodictis olivaris* (AY327324); and *Cranoglanis bouderius* (AF416879).

GenBank accession numbers for outgroup taxa used in the *Cyprinella* dataset are as follows: *Codoma ornata* (GQ275165); *C. analostona* (GQ275236, GQ275237); *C. callisema* (GQ275242); *C. callistia* (GQ275229); *C. callitania* (GQ275238); *C. chloristia* (GQ275227); *C. galactura* (AY249538, AY249539, GQ275224, GQ275225, GQ275226); *C. labrosa* (GQ275182); *C. leedsi* (GQ275239); *C. lutrensis* (GQ275183, GQ275184, GQ275185, GQ 275186, GQ275187, GQ275188); C*. nivea* (GQ275241); *C. rutila* (EU082524); *C. trichoistia* (GQ275245); *C. venusta* (GQ275215); *Dionda (Tampichthys) ipni* (EU082480); and *Pimephales promelas* (GQ275159).
