# Supplementary figures and images for: Comparative Phylogeography of Mississippi Embayment Fishes
Source: PLoS One. 2015 Mar 31;10(3):e0116719. doi: 10.1371/journal.pone.0116719 (PMC4380359; doi:10.1371/journal.pone.0116719)

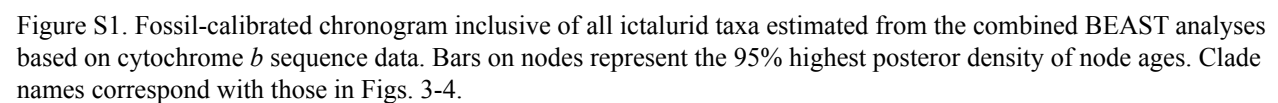

Supplement: S1 Fig — Fossil-calibrated chronogram inclusive of all ictalurid taxa estimated from the combined BEAST analyses based on cytochrome b sequence data. Bars on nodes represent 95% highest posterior density of node ages. Clade names correspond with those in Figs 3 and 4. (PDF) [file pone.0116719.s002.pdf]
