# Supplementary material for: Comparative Phylogeography of Mississippi Embayment Fishes
Source: PLoS One. 2015 Mar 31;10(3):e0116719. doi: 10.1371/journal.pone.0116719 (PMC4380359; doi:10.1371/journal.pone.0116719)
Supplement: S2 Fig — Rate-calibrated chronogram inclusive of all ictalurid taxa estimated from the combined BEAST analyses based on cytochrome b sequence data. Bars on nodes represent 95% highest posterior density of node ages. Clade names correspond with those in Figs 3 and 4. (PDF) [file pone.0116719.s003.pdf]

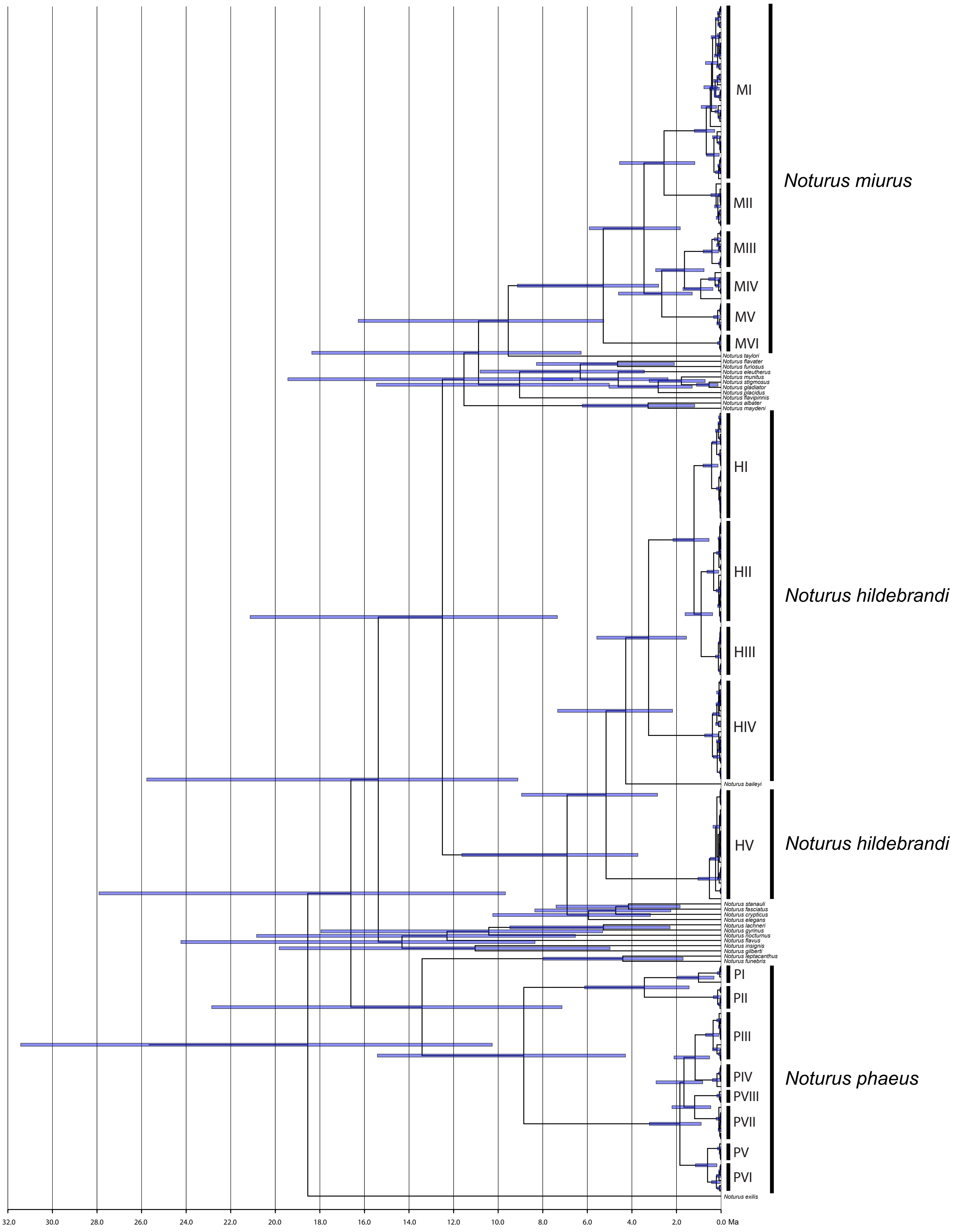

Figure S2. Rate-calibrated chronogram inclusive of all *Noturus* taxa estimated from the combined BEAST analyses based on cytochrome *b* sequence data. Bars on nodes represent the 95% highest posteror density of node ages. Clade names correspond with those in Figs. 3-4.
